# The prognostic role of plasma fibrinogen in adult secondary hemophagocytic lymphohistiocytosis

**DOI:** 10.1186/s13023-020-01622-2

**Published:** 2020-11-25

**Authors:** Guangli Yin, Changfeng Man, Jiayu Huang, Shengen Liao, Xin Gao, Tian Tian, Limin Duan, Ji Xu, Hongxia Qiu

**Affiliations:** 1 Department of Hematology, The First Affiliated Hospital of Nanjing Medical University, Jiangsu Province Hospital, 300 Guangzhou Road, Nanjing, 210029 China; 2 Department of Cardiology, The First Affiliated Hospital of Nanjing Medical University, Jiangsu Province Hospital, 300 Guangzhou Road, Nanjing, 210029 China; 3 Department of Geriatric Hematology, The First Affiliated Hospital of Nanjing Medical University, Jiangsu Province Hospital, 300 Guangzhou Road, Nanjing, 210029 China

**Keywords:** Hemophagocytic lymphohistiocytosis, Fibrinogen, Prognostic, Nonlinear, Mortality

## Abstract

**Background:**

In adult patients with secondary hemophagocytic lymphohistiocytosis (sHLH), no valid immune biomarker has been available for predicting the prognosis of untreated sHLH patients.

**Methods:**

Circulating plasma levels of fibrinogen (FIB) were measured at diagnosis in 293 cases of adult sHLH. We categorized FIB levels into tertiles. Multivariable Cox proportional hazards models were used to evaluate the relationship between FIB and survival. Restricted cubic spline models and two-piecewise Cox proportional hazards models were used to address the nonlinear association between FIB and mortality.

**Results:**

During a median follow-up of 52 (interquartile ranges, 18–221) days, 208 deaths occurred, with 137 deaths in malignancy-associated hemophagocytic lymphohistiocytosis (MHLH) and 71 deaths in non-malignancy-associated hemophagocytic lymphohistiocytosis (non-MHLH). After multivariable adjustment, compared with the highest tertile of FIB, the hazard ratios (HRs) with 95% confidence intervals (CIs) of survival for tertile 2 and tertile 1 were 1.06 (0.90–1.24) and 0.84 (0.71–0.98), respectively. The restricted cubic spline curve displayed a nonlinear and inverse relationship between FIB and mortality. Furthermore, the threshold effect analysis demonstrated that the inflection point for the curve was at an FIB level of 1.76 g/L. The HRs (95% CIs) for survival were 0.68 (0.55–0.83) and 1.08 (0.96–1.21) on the left and right side of the inflection point, respectively.

**Conclusions:**

These results suggest that plasma fibrinogen is nonlinearly and inversely associated with the risk of mortality in adult secondary hemophagocytic lymphohistiocytosis.

## Background

Adult secondary hemophagocytic lymphohistiocytosis (sHLH) is a condition of pathologic immune dysregulation characterized by uncontrolled activation of cytotoxic T lymphocytes and macrophages, producing abnormal hypercytokinemia and leading to multiple organ failure [1]. These abnormal cytokines are responsible for variable clinical symptoms and signs, such as fever, profound pancytopenia, organomegaly (including lymphadenopathy, hepatomegaly, and splenomegaly), liver dysfunction, hypertriglyceridemia, hyperferritinemia, central nervous system (CNS) symptoms, dermatologic abnormalities, and coagulation disorders, that may result in high morbidity and mortality [2]. In the previous studies focusing on outcomes, mortality ranged from 20.4 to 88% depending on the underlying disease and triggering condition, including malignancies, infections, and autoimmune disorders [3]. Early identification of the severity of adult HLH and prognosis would be helpful for enhancing the patients’ chances of survival; therefore, improved prognostic indicators are needed.

Coagulation disorders are common in sHLH patients and are reported in up to 60% of patients with sHLH [4]. Fibrinogen (FIB) less than or equal to 1.50 g/L, one of the most frequently reported coagulopathies and the hallmark of HLH, is also a diagnostic criterion for HLH [5]. Several studies have also demonstrated that 50–80% of sHLH patients have hypofibrinogenemia [6, 7]. Recently, one pediatric HLH study reported that plasma FIB levels (< 150 mg/dL) at diagnosis may be prognostic measures of inferior survival [8]. Several retrospective studies have reported the prognostic role of plasma fibrinogen in adult sHLH [4, 9]. However, these studies were limited by a relatively small sample size (< 120 patients) and lacked follow-up data or adjusted confounding factors influencing prognosis for sHLH patients [9, 10]. In addition, no quantitative analyses were performed to address the dose–response relationship between FIB and the risk of mortality, and whether a linear or nonlinear relationship exists between FIB and the risk of mortality is still unknown.

Therefore, whether reduced FIB levels are associated with poor survival in adult sHLH independent of conventional risk factors currently remains unclear. Thus, we aimed to explore whether reduced FIB levels independently affected long-term survival in a retrospective cohort study with sHLH.

## Methods

### Study patients

**Fig. 1:**
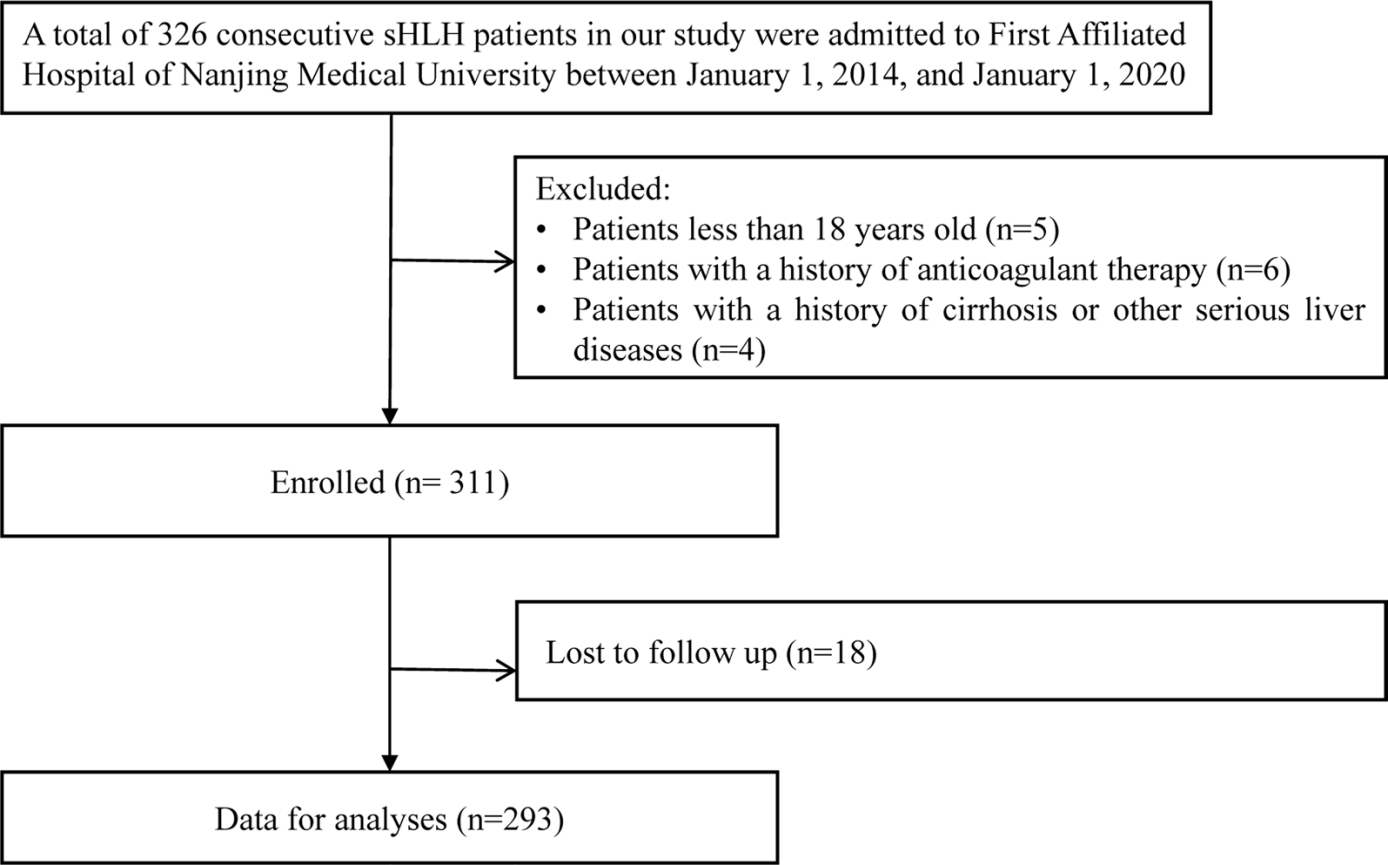
Study flow chart. *sHLH* secondary hemophagocytic lymphohistiocytosis

A total of 293 consecutive patients with a primary diagnosis of adult secondary hemophagocytic lymphohistiocytosis (sHLH) between January 1, 2014, and January 1, 2020, were enrolled. The HLH-2004 criteria developed by Henter et al. [11] and the HScore by Fardet et al. [12] were routinely used to help with the diagnosis of HLH. The inclusion of patients was based on (1) age ≥ 18 years and (2) fulfillment of at least 5 of the 8 criteria proposed by the Histiocyte Society in 2004. The exclusion criteria included (1) Patients less than 18 years old (n = 5); (2) patients with a history of anticoagulant therapy (n = 6); and (3) patients with a history of cirrhosis or other serious liver diseases (n = 4). Figure 1 shows the flow chart of the patient inclusion and exclusion process in this study. Our study was approved by the ethics committee of the First Affiliated Hospital of Nanjing Medical University (Clinical Trial: ChiCTR2000032421). The study was conducted in accordance with the Declaration of Helsinki. Informed consent was obtained to review patient medical records.

### Clinical data

Patients’ histories and clinical characteristics including age; gender; fever; complete blood cell counts (CBC); hepatosplenomegaly; and blood biochemical tests (including aspartate aminotransferase (AST), alanine aminotransferase (ALT), triglycerides (TG), lactate dehydrogenase (LDH), fibrinogen (FIB), ferritin, serum soluble interleukin-2 receptor (sIL-2R, sCD25), and β_2_-microglobulin (β_2_-MG) tests) were reviewed from their medical records on admission. Epstein-Barr virus (EBV) was evaluated by both serology and EBV DNA real-time quantitative polymerase chain reaction (RQ-PCR) analysis. The NK-cell cytotoxicity assay is not available at our facility. Bone marrow aspiration and biopsy samples were reviewed at the first diagnosis. Tumor or lymph node biopsy and PET/CT confirmed malignancy-associated hemophagocytic lymphohistiocytosis (MHLH).

The FIB levels were measured in the clinical laboratory of our hospital following regular procedures and assayed by the Clauss method with an automatic coagulometer (Sysmex CS5100, Japan). The FIB levels were categorized in tertiles according to the distributions of the study population: tertile 1 (T1) ≤ 1.20, 1.21 ≤ tertile 2 (T2) ≤ 1.97 and tertile 3 (T3) > 1.97.

### Follow-up and endpoints

The primary outcome of the current study was overall survival (OS), which was calculated as the time in days from sHLH diagnosis to the date of death from any cause or the last follow-up. The survival status of all participants was confirmed with death records or a telephone call to the patient’s relatives or to the patient themselves and ascertained by checking government records of death.

### Treatment according to causes of sHLH

Two hundred and seventy-eight patients (95.2%) received a specific treatment for sHLH. In our 169 MHLH patients, 120 patients had received systemic combination chemotherapy as the first-line therapy for sHLH; 33 patients were treated with HLH-94 as the initial therapy; 10 patients with progressive multiple organ dysfunction were treated with only intravenous immunoglobulins (IVIg) and glucocorticoid (GC). In our 124 non-malignancy-associated hemophagocytic lymphohistiocytosis (non-MHLH) patients, first-line treatment using HLH-94 was administered in 14 patients; GC was administered in 11 patients; GC + IVIg was administered in 46 patients; GC + etoposide was administered in 26 patients; GC + IVIg + cyclosporine was administered in 15 patients; and GS + IVIg + cyclophosphamide was administered in 4 patients. No difference in treatment regimens occurred across the FIB groups.

### Statistical analysis

Continuous variables were presented as the means with standard deviations (SDs) for normally distributed variables or the median with the interquartile range (IQR) for nonnormally distributed variables, and categorical variables were described as numbers and proportions (percentages). Differences across FIB tertiles were analyzed using one-way ANOVA and the Kruskal–Wallis and chi-squared tests where appropriate. We used univariate and multivariable Cox proportional hazards models to estimate hazard ratios (HRs) and 95% CIs to examine the relationship between fibrinogen concentrations and survival (variables with *P* < 0.05 in the univariate Cox regression were subsequently entered into the multivariable model). The fibrinogen of T3 was used as a reference to estimate HR and 95% CI. Kaplan–Meier curves for tertiles of fibrinogen were plotted to show mortality rates. Survival curves were adjusted for covariates derived from the final model according to multivariable Cox proportional hazards models.

A restricted cubic spline (with 3 knots located at the 10th, 50th, and 90th percentiles; the number of knots was selected according to the Akaike information criterion) was conducted to address the nonlinear relationship between fibrinogen and the risk of mortality after adjusting for confounding factors. If nonlinearity was detected, we calculated the inflection point using a recursive algorithm and then constructed a two-piecewise Cox proportional hazards model on both sides of the inflection point. The threshold level was calculated according to the inflection point with the maximum model likelihood using the trial and error method. A log likelihood ratio test comparing the one-line Cox proportional hazards model with a two-piecewise Cox proportional hazards model was performed to examine the statistical significance.

Stratified analyses were conducted according to gender (male or female), age (≤ 60 years or > 60 years), ferritin (≤ 10,000 µg/L or > 10,000 µg/L), sCD25 (≤ 20,000 ng/L or > 20,000 ng/L), EBV status (no EBV infection or EBV infection), and etiologies (MHLH or non-MHLH). We tested for potential effect modification by these stratification variables by including interaction terms between fibrinogen and a potential effect modifier (overall survival) in the multivariate adjusted model and by conducting a likelihood ratio test (LRT) comparing the models with and without interaction terms. Tests for trends were calculated by including the median value for each corresponding tertile as a continuous variable in the models [13]. All statistical analyses were conducted with R software (version 3.6.0; The R Foundation for Statistical Computing) and STATA statistical software (version 14.0; StataCorp, TX, USA). A two-sided *P* < 0.05 was considered statistically significant for all analyses.

## Results

### Distribution of plasma fibrinogen and patient characteristics

The baseline characteristics for patients stratified by FIB tertiles are listed in Table 1. The mean age of the total study population was 53 (41–64) years, and 62.5% were male. The median FIB level in the overall population was 1.50 g/L (IQR 1.02–2.18 g/L). The participants with lower FIB had lower platelet levels and higher ALT, AST, LDH, TG, β_2_-MG, serum ferritin, and sCD25 levels and total Hscore points and were more likely to be infected with EBV (all *P* < 0.05). Detailed etiological classification of sHLH patients was shown in Additional file 1.

**Table 1:** Baseline demographic, clinical, and laboratory characteristics of the study patients according to tertiles of fibrinogen

	Total (N = 293)	Tertile 1 (N = 99)	Tertile 2 (N = 97)	Tertile 3 (N = 97)	*P*
FIB, g/L	1.50 (1.02–2.18)	0.86 (0.70–1.03)	1.51 (1.38–1.72)	2.59 (2.18–3.33)	*< 0.001*
Male, n (%)	183 (62.5)	62 (62.6)	60 (61.9)	61 (62.9)	0.988
Age, years	53 (41–64)	51 (33–64)	56 (39–62)	55 (46–66)	0.118
ANC, $\times \hspace{0.17em}$10^9^/L	1.32 (0.69–2.35)	1.04 (0.57–2.11)	1.36 (0.77–2.24)	1.55 (0.81–2.71)	0.057
HB, g/L	86 (71–100)	84 (77–95)	90 (73–107)	82 (68–97)	0.111
PLT,$\times \hspace{0.17em}$10^9^/L	43 (24–72)	31 (18–52)	45 (25–80)	54 (32–97)	*< 0.001*
ALT, U/L	67.1 (36.3–149.1)	98.6 (50.6–235.4)	77.6 (32.4–143.2)	50.1 (28.4–82.3)	*< 0.001*
AST, U/L	91 (46.1–203.8)	178.5 (78.1–411.0)	90.2 (45.1–198.1)	61.0 (39.5–121.65)	*< 0.001*
LDH, U/L	676 (394.0–1308.5)	1004 (580.0–1793.0)	646 (354.5–1101.0)	540 (379.5–934.0)	*< 0.001*
TG, mmol/L	2.47 (1.71–3.70)	2.78 (1.77–4.04)	2.52 (1.89–3.67)	2.01 (1.58–3.18)	*0.015*
Ferritin, ug/L	4739 (1522–12,798)	8133 (2000–20,000)	3639 (1613–9558)	2841 (1494–7095)	*< 0.001*
sCD25, ng/L	33,480 (17,442–51,668)	40,042 (22,660–55,919)	26,920 (14,266–48,289)	32,939 (16,018–47,762)	*0.024*
*β*_*2*_-MG, mg/L	5.97 (4.41–8.53)	5.91 (4.19–8.53)	7.63 (4.85–9.28)	5.69 (3.88–8.04)	*0.030*
Fever, °C	39.5 (39.0–40.0)	39.6 (39.0–40.0)	39.4 (39.0–40.0)	39.3 (39.0–40.0)	0.143
Splenomegaly (%)	260 (88.7)	90 (90.9)	88 (90.7)	82 (84.5)	0.278
Lymphadenopathy (%)	152 (51.9)	45 (45.5)	52 (53.6)	55 (56.7)	0.265
Hemophagocytic (%)	260 (88.7)	88 (88.9)	85 (87.6)	87 (89.7)	0.900
HScore, points	234 (205–269)	255 (224–283)	235 (210–274)	219 (179–244)	*< 0.001*
EBV infection (%)	148 (50.5)	65 (65.7)	44 (45.4)	39 (40.2)	*0.001*
Etiology					0.120
MHLH (%)	169 (57.7)	65 (65.7)	50 (51.5)	54 (55.7)	
Non-MHLH (%)	124 (42.3)	34 (34.3)	47 (48.5)	43 (44.3)	
Treatment					0.960
Chem ± HLH-94 (%)	167 (57.0)	58 (58.6)	53 (54.6)	56 (57.7)	
GC ± IVIG ± CsA ± VP16 (%)	112 (38.2)	36 (36.4)	40 (41.2)	36 (37.1)	
Support (%)	14 (4.8)	5 (5.1)	4 (4.1)	5 (5.2)	

Italics indicate statistical significance (*P* < 0.05)

*FIB* fibrinogen, *ANC* absolute neutrophil count, *HB* hemoglobin, *PLT* platelet, *ALT* alanine transaminase, *AST* aspartate transaminase, *LDH* lactate dehydrogenase, *ALB* albumin, *TG* triglyceride, *sCD25* soluble interleukin-2 receptor, *β*_*2*_*-MG* beta_2_-microglobulin, *EBV* Epstein-Barr virus, *MHLH* malignancy-associated haemophagocytic lymphohistiocytosis, *Non-MHLH* non-malignancy associated haemophagocytic lymphohistiocytosis, *GC* glucocorticoid, *IVIg* intravenous immunoglobulins, *CsA* cyclosporine, *VP16* etoposide

### Associations of the plasma fibrinogen with survival

**Fig. 2:**
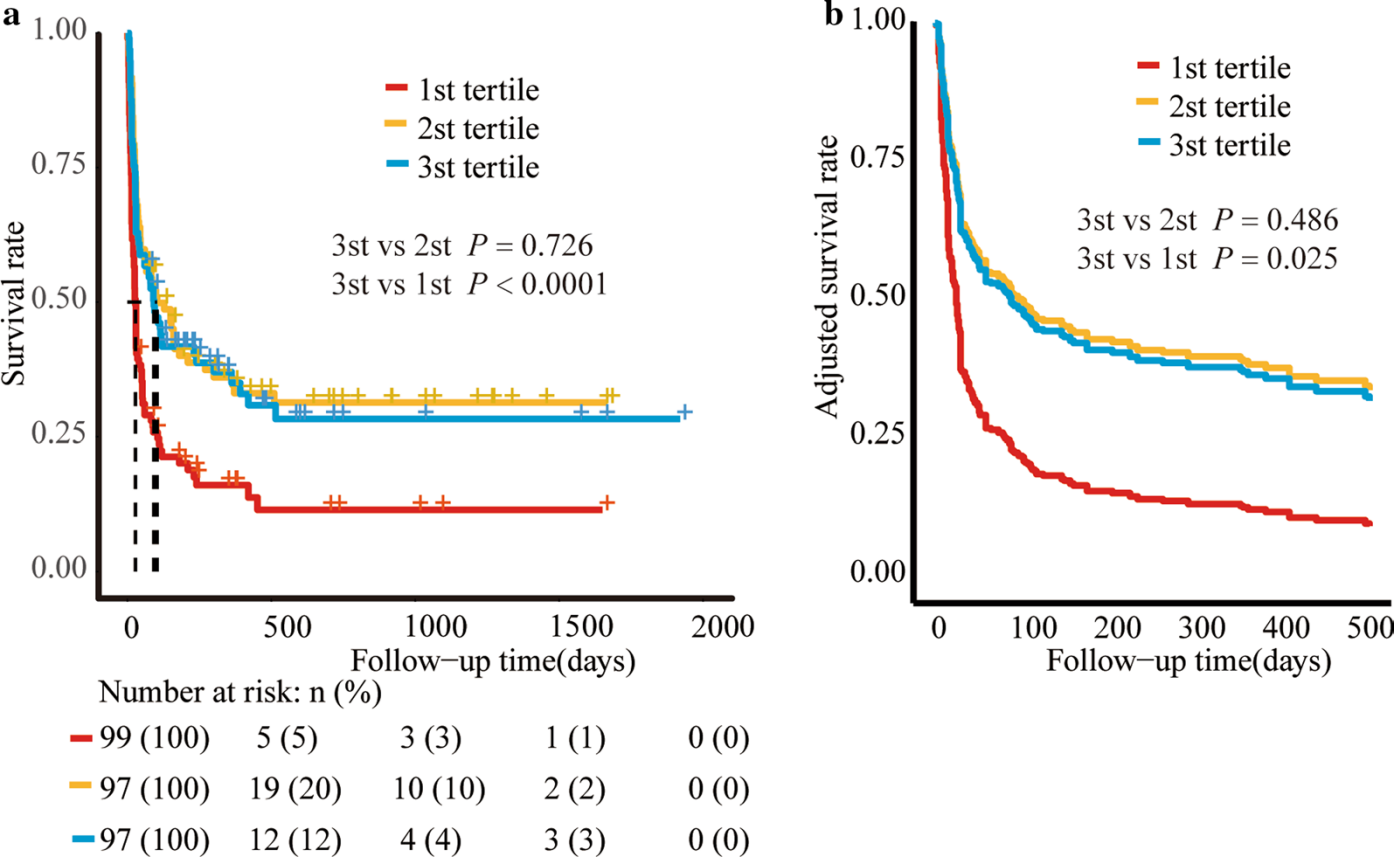
Survival curves of all sHLH patients according to fibrinogen tertiles. **a** Kaplan–Meier survival curves. **b**, Adjusted survival curves: curves were adjusted for age, PLT (platelet), EBV, ferritin, malignancy-associated HLH (MHLH) and treatment strategies

**Fig. 3:**
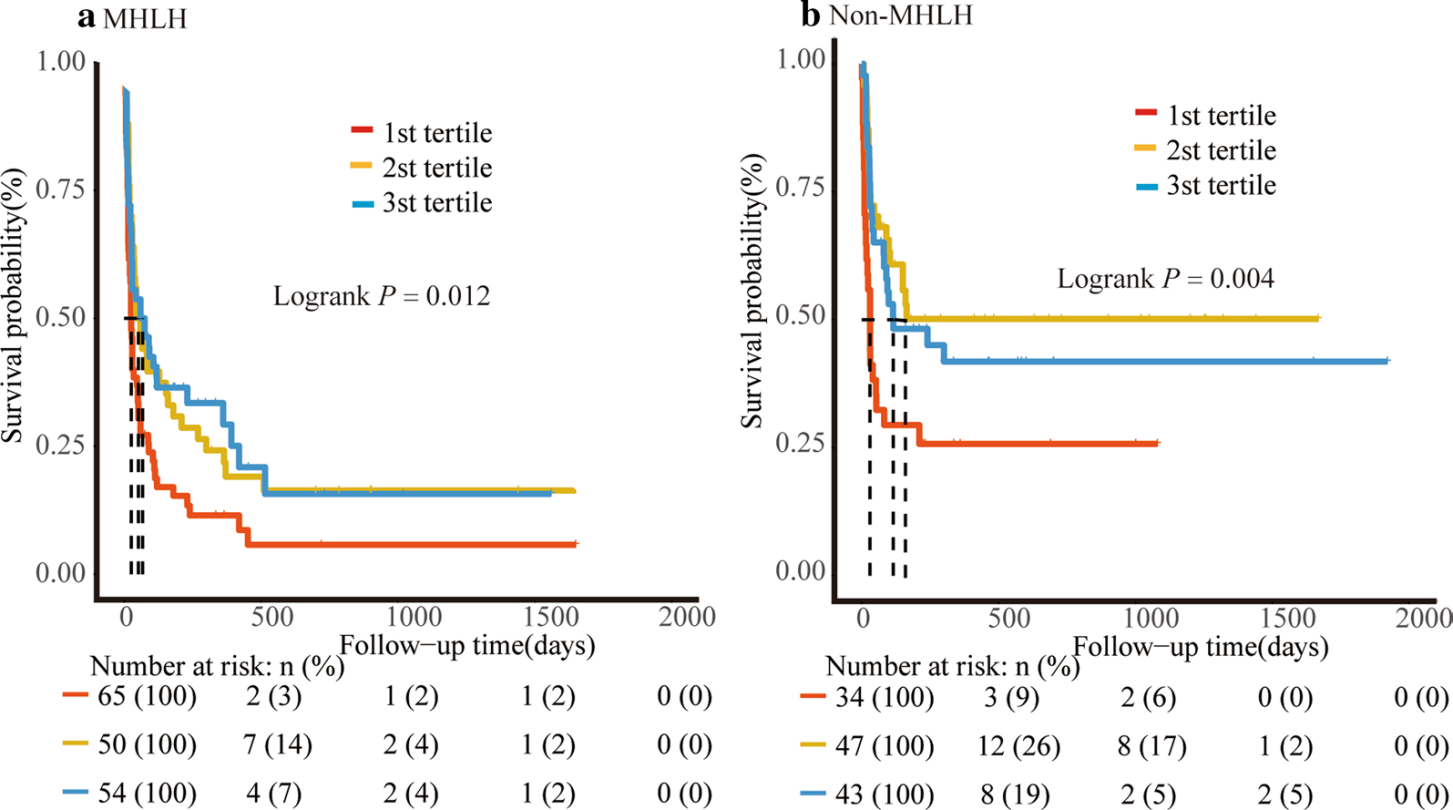
Survival curves of sHLH patients according to fibrinogen tertiles. **a** MHLH; **b** non-MHLH. *MHLH* malignancy-associated HLH, *non-MHLH* non-malignancy-associated hemophagocytic lymphohistiocytosis

The median follow-up duration was 52 (IQR, 18–221) days. The follow-up found 208 deaths, including 137 deaths in MHLH and 71 deaths in non-MHLH. Figure 2 shows the survival curves with regard to different categories of FIB in the total patients for cumulative overall survival. Patients with T3 and T2 had a similar survival (*P* = 0.726), whereas patients with T1 had a significantly worse survival (HR = 0.76, 95% CI 0.66–0.87; *P*<0.001) than those with T3. After adjusting for confounding factors, including age, PLT, ferritin, EBV infection, MHLH, and treatment remedies, the HRs with 95% CIs of survival for T2 and T1 were 1.06 (0.90–1.24) and 0.84 (0.71–0.98), respectively (*P* for trend: 0.04) (Table 2 and Fig. 2). This finding was also in line with MHLH and non-MHLH group (Fig. 3).

**Table 2:** Uni- and multi-variate Cox regression analyses of survival

Characteristics	Unadjusted		Adjusted	
	HR (95% CI)	*P*	HR (95% CI)	*P*
Male	0.85 (0.75–0.96)	*0.010*		
Age, years	1.00 (0.99–1.00)	*0.009*	1.00 (0.99–1.00)	*0.017*
ANC > 1.0 × 109/L	0.94 (0.84–1.06)	0.331		
HB < 90 g/L	0.87 (0.77–0.98)	*0.027*		
PLT < 100 × 10^9^/L	0.76 (0.62–0.91)	*0.004*	0.81 (0.66–0.99)	*0.043*
LDH ≥ 2.5 × ULN	1.14 (0.93–1.39)	0.205		
TG ≥ 3.0 mmol/L	0.93 (0.82–1.05)	0.245		
Fever, ≥ 39.1 °C	1.02 (0.90–1.15)	0.775		
Splenomegaly	0.95 (0.78–1.16)	0.636		
Hemophagocytosis	1.08 (0.9–1.29)	0.403		
Log_10_ (Ferritin)	0.75 (0.66–0.85)	*< 0.001*	0.78 (0.68–0.90)	*0.001*
Log_10_ (sCD25)	0.77 (0.65–0.92)	*0.003*		
EBV infection	0.78 (0.69–0.88)	*< 0.001*	0.85 (0.74–0.96)	*0.011*
MHLH	0.78 (0.69–0.88)	*< 0.001*	0.63 (0.54–0.74)	*< 0.001*
Treatment				
Support treatment	1.00 (Ref.)		1.00 (Ref.)	
GC + IVIG	0.90 (0.73–1.11)	0.323	1.08 (0.87–1.35)	0.462
Chem ± HLH94	1.15 (0.93–1.40)	0.196	1.74 (1.38–2.22)	*< 0.001*
FIB(g/L)				
3 st tertile	1.00 (Ref.)		1.00 (Ref.)	
2 st tertile	1.03 (0.88–1.20)	0.726	1.06 (0.90–1.24)	0.486
1 st tertile	0.76 (0.66–0.87)	*< 0.001*	0.84 (0.71–0.98)	*0.025*

Italics indicate statistical significance (*P* < 0.05)

*ANC* absolute neutrophil count, *HB* hemoglobin, *PLT* platelet, *LDH* lactate dehydrogenase, *TG* triglyceride, *sCD25* soluble interleukin-2 receptor, *EBV* Epstein-Barr virus, *MHLH* malignancy-associated haemophagocytic lymphohistiocytosis, *GC* glucocorticoid, *IVIg* intravenous immunoglobulins, *FIB* fibrinogen, *HR* hazard ratio, *CI* confidence interval

**Fig. 4:**
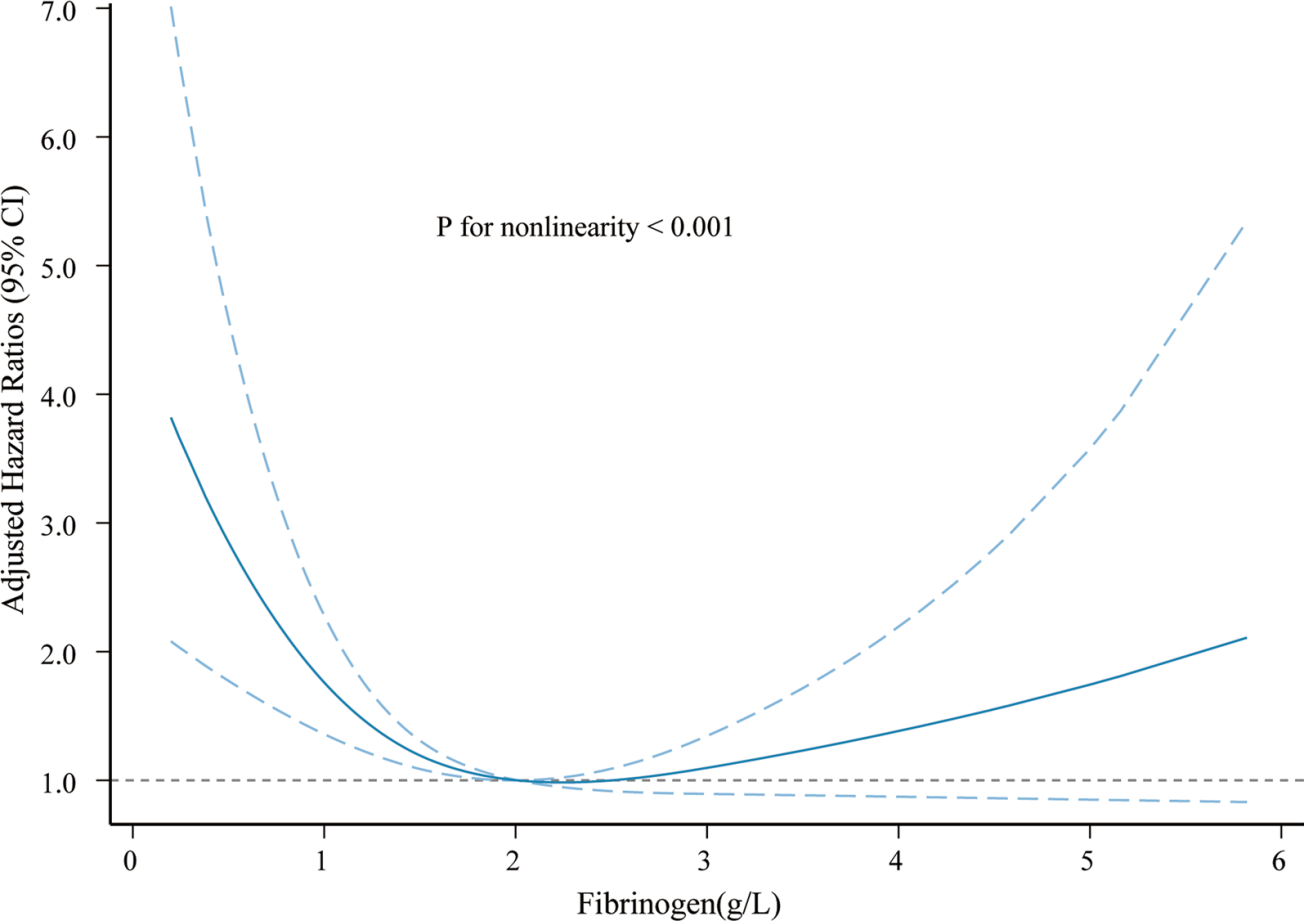
Cubic spline plot of the association between plasma fibrinogen and the risk of mortality among adult onset HLH patients. The solid line and dashed line represent the estimated hazard ratios and their corresponding 95% CIs, respectively. Analyses were adjusted for age, PLT (platelet), EBV, ferritin, malignancy—associated HLH (MHLH) and treatment strategies

The associations between FIB (continuously) and the risk of mortality are shown in Fig. 4. The fully adjusted cubic curve fitting showed that FIB was nonlinearly and inversely associated with the risk of death, and the tests for nonlinearity were significant (*P* for nonlinearity < 0.001). We further conducted a threshold effect analysis of FIB on the survival. When we fitted the association between FIB and survival using a Cox proportional hazards model and a two-piecewise Cox proportional hazards model, the *P* value for the log likelihood ratio test was less than 0.001, indicating that the two-piecewise Cox proportional hazards model was more suitable for fitting the association between FIB and survival. The inflection point that we identified for FIB was 1.76 g/L. When FIB was ≤ 1.76 g/L, decreased FIB was significantly associated with an increased risk of poor survival (HR = 0.68, 95% CI, 0.55–0.83; *P *< 0.001). Conversely, when FIB was > 1.76 g/L, the survival did not change significantly with increased FIB (HR = 1.08, 95% CI, 0.96–1.21; *P* = 0.187) (Table 3).

**Table 3:** Threshold effect analysis of fibrinogen on survival using two-piecewise Cox proportional hazards regression models

Inflection point	Group	HR (95% CI)	*P* value	*P* for log likelihood ratio test
1.76	≤ 1.76	0.68 (0.55–0.83)	< 0.001	< 0.001
	> 1.76	1.08 (0.96–1.21)	0.187	

Analyses were adjusted for age, PLT (platelet), EBV, log10-transform ferritin, MHLH and treatment strategies

*HR* hazard ratio, *CI* confidence interval

### Subgroup analysis

The subgroup analyses on survival according to multiple confounding variables are shown in Table 4. Although no significant interactions for the associations between FIB and survival were observed across strata by age, sex, ferritin, sCD25, EBV infection and etiologies, participants who were younger, who were male, who had ferritin ≤ 10,000 µg/L, who had a high level of sCD25, who had EBV infection, and who were non-MHLH were more likely to have a worse survival with a decrease in FIB.

**Table 4:** Stratified associations between fibrinogen and survival

Subgroups	Tertile 3	Tertile 2	Tertile 1	*P* for trend	*P* for interaction
	HR (95% CI)	HR (95% CI)	HR (95% CI)		
Gender					0.800
Male	1.00 (Ref)	1.02 (0.84–1.25)	0.80 (0.65–0.98)*	0.068	
Female	1.00 (Ref)	1.14 (0.86–1.51)	0.86 (0.65–1.14)	0.458	
Age ≤ 60 years					0.478
Yes	1.00 (Ref)	1.00 (0.79–1.28)	0.82 (0.65–1.04)	0.172	
No	1.00 (Ref)	1.13 (0.91–1.39)	0.90 (0.73–1.12)	0.480	
Ferritin > 10,000 ug/L					0.855
Yes	1.00 (Ref)	0.91 (0.64–1.29)	0.89 (0.64–1.24)	0.499	
No	1.00 (Ref)	1.12 (0.94–1.34)	0.80 (0.67–0.97)*	0.140	
sCD25 > 20,000 ug/L					0.510
Yes	1.00 (Ref)	0.93 (0.77–1.12)	0.81 (0.67–0.97)*	*0.030*	
No	1.00 (Ref)	1.49 (1.09–2.06)*	0.97 (0.71–1.34)	0.604	
EBV infection					0.905
Yes	1.00 (Ref)	0.97 (0.78–1.21)	0.79 (0.64–0.99)*	0.057	
No	1.00 (Ref)	1.20 (0.95–1.51)	0.86 (0.67–1.08)	0.531	
MHLH					0.171
Yes	1.00 (Ref)	1.06 (0.87–1.29)	0.90 (0.75–1.10)	0.378	
No	1.00 (Ref)	1.06 (0.82–1.39)	0.72 (0.55–0.96)*	0.080	

Analyses were adjusted for age, PLT (platelet), EBV, log_10_-transform ferritin, MHLH and treatment strategies when they were not stratified variables

Italics indicate statistical significance (*P* < 0.05)

*HR* hazard ratio, *CI* confidence interval

*P < 0.05; **P < 0.001

## Discussion

To our knowledge, this is the largest cohort study assessing the prognostic role of fibrinogen in adult sHLH. In addition, our study first indicated that plasma fibrinogen level is nonlinearly and inversely associated with poor survival in adult sHLH patients regardless of conventional confounding factors, including age, PLT, EBV infection, ferritin, etiologies and treatment strategies. Further threshold effect analysis showed that the inflection point was 1.76 g/L, and survival was inconsistent on the left and right sides of the inflection point.

A few previous studies regarding the association of FIB and its threshold with the risk of mortality defined plasma FIB levels using different categories or cut-off values. In pediatric patients with HLH [8, 14], Signoff demonstrated that hypofibrinogenemia (FIB < 1.5 g/L) was independently associated with higher mortality rates (adjusted odds ratio, 6.0; 95% CI, 2.0–18.1); meanwhile, in adult sHLH studies, Sandrine et al. [4] used 117 ICU patients and found that coagulation disorders were associated with higher mortality, especially FIB < 2 g/L (adjusted OR = 2.42, 95% CI 1.08–5.41; *P* = 0.04). The large sample size of this study combined with the application of RCS afforded us greater statistical power and model flexibility to characterize the associations of plasma FIB concentrations with mortality.

The increased risk of mortality associated with low fibrinogen might be ascribed to the activation of lymphocyte and macrophage immune cells, creating an uncontrolled loop of inflammation that is responsible for liver dysfunction, DIC and fibrinolysis. Stimulated macrophages secrete proinflammatory cytokines, including TNF alpha, IL-1beta, and IL-6, which in turn can release tissue plasminogen activator in excess; correspondingly, an increase in plasmin responsible for fibrinolysis leads to low fibrinogen levels [15, 16]. In addition, high IFN-γ, presented in sHLH, can induce the production of tissue factor expression in activated macrophages, and activated histiocytes can activate factor X through Mac-1 receptors, eventually initiating blood coagulation and aberrantly triggering an overconsumption of fibrinogen [17, 18]. Another mechanism could be diffuse liver infiltration with activated T lymphocytes and macrophages and hypercytokinemia [19]. All of the above mechanisms, alone or together, may be attributed to low fibrinogen levels, which reflect low in vivo disease activity. Hypofibrinogenemia was also indicative of adverse outcomes beyond coagulopathy, as indicated by its persistent association with a complicated course and sHLH/MAS with hyperinflammation.

Our study explored the nonlinear association between plasma FIB levels and the risk of mortality and indicated that FIB displayed an L-shaped relationship with the risk of mortality. One hypothesis is that the lower fibrinogen level (≤ 1.76 g/L) is only the reflection of a more severe form of HLH (with more intense cytokine storm and intense hemophagocytic activity). These patients with lower fibrinogen levels who have worse survival may reach a certain threshold of excessive activated immune cells and overwhelming systemic inflammation. Early identification of these patients may prompt earlier consideration of alternative therapeutic strategies, including intensive immunotherapies.

Our study has several strengths. This is the first study to address the nonlinearity between FIB levels at the diagnosis of adult sHLH and the risk of mortality and further explains this nonlinearity with a threshold effect analysis. However, several limitations of our study were noted. First, due to the single-center retrospective study, the results may not be generalizable to a broader population. Second, although we had fully adjusted a broad set of covariates available to influence prognosis, we could not rule out the possibility that our findings were biased by unmeasured or unrecognized confounders. Finally, the observational study could only demonstrate the association between FIB levels and the prognosis of patients with sHLH; it could not provide conclusions regarding causality.

## Conclusion

Our study demonstrated that plasma FIB levels are nonlinearly and inversely associated with poor survival in adult sHLH patients. The threshold effect of FIB on poor survival was 1.76 g/L. Each 1 unit decrease in FIB was associated with a 32% decrease in survival when the serum FIB levels were ≤ 1.76 g/L.

## Supplementary information

**Additional file 1**. Detailed etiological classification of 293 adult sHLH (MHLH and Non-MHLH).

## Data Availability

The data of our patients is available in the Department of Medical Records at Jiangsu Province Hospital and the First Affiliated Hospital of Nanjing Medical University. These data can be released with consent from the patients and are available from the corresponding author upon reasonable request.

## Abbreviations

sHLH: Secondary hemophagocytic lymphohistiocytosis
FIB: Fibrinogen
ANC: Absolute neutrophil count
HB: Hemoglobin
PLT: Platelet
ALT: Alanine transaminase
AST: Aspartate transaminase
LDH: Lactate dehydrogenase
TG: Triglyceride
sCD25: Soluble interleukin-2 receptor
β_2_-MG: Beta_2_-microglobulin
EBV: Epstein-Barr virus
MHLH: Malignancy-associated hemophagocytic lymphohistiocytosis
Non-MHLH: Non-malignancy associated hemophagocytic lymphohistiocytosis
GC: Glucocorticoid
IVIg: Intravenous immunoglobulins
CsA: Cyclosporine
VP16: Etoposide
HR: Hazards ratio
95% CI: 95% Confidence interval
