# Supplementary material for: The prognostic role of plasma fibrinogen in adult secondary hemophagocytic lymphohistiocytosis
Source: Orphanet J Rare Dis. 2020 Nov 25;15:332. doi: 10.1186/s13023-020-01622-2 (PMC7690012; doi:10.1186/s13023-020-01622-2)
Supplement: Supplementary file 1 — Additional file 1. Detailed etiological classification of 293 adult sHLH (MHLH and Non-MHLH). [file 13023_2020_1622_MOESM1_ESM.docx]

Additional file 1. Detailed etiological classification of 293 adult sHLH (MHLH and Non-MHLH).

| Etiologies | Number (%) |
| --- | --- |
| **Non-MHLH** | 124 (42.3%) |
| **Infection-associated HLH** | 69 (23.5) |
| EBV | 51 |
| CMV | 3 |
| HIV | 2 |
| Staphylococcus sepsis | 8 |
| Pulmonary infection | 5 |
| **Autoimmune disorder associated HLH** | 21(7.2) |
| SLE | 8 |
| AOSD | 8 |
| SS | 3 |
| UCTD | 2 |
| **Unknown cause** | 34 (11.6) |
|  |  |
| **MHLH** | 169 (57.7) |
| T cell lymphoma |  |
| NK/T cell lymphoma | 49 |
| Peripheral T cell lymphoma, NOS | 8 |
| Anaplastic large cell lymphoma | 2 |
| Angioimmunoblastic T cell lymphoma | 4 |
| Unclassified | 33 |
| B cell lymphoma |  |
| Diffuse large B cell lymphoma | 17 |
| Intravascular large B-cell lymphoma | 5 |
| Chronic lymphoblastic leukemia/  small lymphocytic lymphoma | 1 |
| Burkitt lymphoma | 3 |
| Unclassified | 40 |
| Myelodysplastic syndromes with excess blasts | 1 |
| Hodgkin’s lymphoma | 2 |
| Acute leukemia | 4 |

sHLH, secondary hemophagocytic lymphohistiocytosis; Non-MHLH, non-malignancy associated HLH; MHLH, malignancy-associated HLH; EBV, Epstein-Barr virus; CMV, Cytomegalovirus; HIV, Human immunodeficiency virus; SLE, Systemic lupus erythematosus; AOSD, Adult onset Still’s disease; SS, Sjogren’s syndrome; UCTD, Undifferentiated connective tissue disease.
